# Intrinsic roles in the crosshair – strategic analysis of multi-site role implementation with an adapted matrix map approach

**DOI:** 10.1186/s12909-019-1628-5

**Published:** 2019-06-27

**Authors:** Jan Griewatz, Maria Lammerding-Koeppel

**Affiliations:** Eberhard-Karls University of Tuebingen, Competence Centre for University Teaching in Medicine, Baden-Wuerttemberg, Elfriede-Aulhorn-Str. 10, 72076 Tuebingen, Germany

**Keywords:** Undergraduate medical education, Competence orientation, CBME framework, NKLM, Intrinsic roles, Implicit-explicit relation, Curricular transparency, Curriculum development, Curriculum mapping, Matrix map method

## Abstract

**Background:**

The implementation of competency-based intrinsic roles in undergraduate medical education remains a challenge. Faculties in transition need to be provided with generalizable curricular data in order to facilitate orientation on curricular roles’ representation and to decide on steps of curriculum development. Explicit and implicit representation of objectives and multi-site agreement can be viewed as status indicators for the adoption of roles. Our aim was to develop a pragmatic cross-locational approach to capture roles’ developmental status in an overview and prioritize strategic recommendations.

**Methods:**

Based on the mapping data from six German medical faculties, the relationship between explicit and implicit curricular representation of role’ objectives (weighting) and extent of programs’ consent (agreement) was calculated. Data was visualized in a role-specific Matrix Map to analyse roles’ implicit-explicit relation and risk-value potential. The matrix was combined with Roger’s stages of innovation diffusion for differentiated interpretation of the developmental role status.

**Results:**

Entangling multi-site agreement and curricular weighting, the 4-Field-Matrix allows to assess objectives based on their current localization in a quadrant: “Disregard” (lower left) and “Progress” quadrant (upper left) reveal the diffusion period; “Potential” (lower right) and “Emphasis” quadrant (upper right) indicate the adoption period. The role patterns differ in curricular representation, progression and clarity: (1) Scholar: explicit/implicit - scattered across the matrix; most explicit objectives in “Progress”. (2) Health Advocate: explicit – primarily in “Emphasis”; only role in which the explicit representation significantly exceeds the implicit. (3) Collaborator: explicit - mainly “Potential”; implicit - “Progress” or “Emphasis”. (4) Professional: explicit – primarily “Potential” but also “Emphasis”; implicit - “Progress” and “Emphasis”; appears better adopted but scattered in weighting; high hidden curricula. (5) Manager: explicit and implicit - exclusively in “Potential”, without signs of development. Role patterns correspond to evidences from literature. Exemplified with roles, quadrant-specific strategies and measures are suggested. Framework reviewers may gain information for discussion of critical content.

**Conclusion:**

The Matrix Map enables to catch intuitively the status of intrinsic roles’ profiles regarding role pattern, implicit-explicit relation and programs agreement. Thus, interpretation and informed discussions are fostered. Further target-oriented analyses and strategic developments can be conducted to enhance transparency and resource-efficiency.

## Background

Worldwide, national frameworks for competency-based medical education (CBME) elaborate the concept of professional roles of the medical doctor as overarching structure, to foster scientifically grounded holistic patientcare. The role concept clarifies the diverse facets of typical medical tasks. Thus, it must be explicitly defined as an integral component in combination with medical content and context. The Medical Expert as the central and integrative role overlaps with competencies that are also described in the six peripheral, so-called “intrinsic roles” [1]: Communicator, Collaborator, Manager, Professional, Health Advocate, and Scholar [1–4]. The professional role concept is internationally widely accepted by medical teachers who rated its relevance in practice high [5–7]. However, even decades after implementation, medical faculties are still challenged to adequately establish these intrinsic roles with explicit transparency in curricula and teaching practice [8, 9]. International studies indicate considerable discrepancies between official well-elaborated frameworks, framework perception and sobering curricular practice from perspectives of teachers, supervisors and students [5, 7, 10–15].

This situation gives serious concern to quality management of programs for several reasons: First, intrinsic role transparency has a direct impact on program success. Effective teaching and sustainable learning require clear labelling and explicit integration of appropriate competencies in core medical training to enhance commitment, predictability and common understanding for assessment [16–20]. Second, outside the explicit curriculum, effective learning also takes place in the hidden curriculum (often called informal or implicit). This implicit learning is individual, variable, often unplanned in learning opportunities whenever they may arise in clinical or non-clinical settings [18, 21–23]. It has proven relevant too and is most likely supportive, as far as is convergent to the explicit curriculum [18, 22], but it is beyond curricular control. The above findings support a strong demand for greater critical awareness towards the explicit and implicit curricula indicating the status of the roles’ adoption [18, 23]. Cross-locational data promises to provide curriculum developers and quality managers with guidance on the general curricular dissemination of role content and the extent of faculty agreement.

In Germany, a cross-site approach for monitoring curricular development was designed using the example of intrinsic roles. Germany is in an early stage of transforming undergraduate medical education (UME) to CBME. In 2015, the deans of all German medical faculties officially adopted the National Competency-based Learning Objectives for Undergraduate Medical Education (NKLM) in its first version, consenting to review the catalogue until 2020 [24, 25]. Thus, the German situation may serve as an example of how to monitor framework implementation. Faculties in transition need to be continuously provided with (self-) evaluative curricular data [23]. Curriculum mapping promises transparency [26] but produces large amounts of data with increased complexity. Additional concepts are helpful to strike the best balance between the demands for pragmatism in analyses as well as for adequate accuracy in fine-tuning curricular requirements. Meaningful, generalizable data may facilitate interpretation of curricular roles’ presentation and prioritizing decisions on curriculum development.

In the present study comprehensive mapping data from six faculties were included. Two approaches derived from economics, business administration and social sciences were applied to provide a basis for systematic visualization and interpretation of roles’ curricular depiction: a Matrix Map Method [27–29] combined with Rogers’ theoretic model of innovation diffusion [28]. As a qualitative visual tool, an adapted 4-Field-Matrix can display complex relations in a simplified overview. In this Matrix Map the explicit and implicit curricular weighting of the intrinsic roles and the extent of programs’ agreement can be interlaced in a single, compelling image. Thus, the 4-Field-Matrix promises a helpful starting point but provides only a relatively rough picture. Adding the ideas of Rogers theory offers the chance to get more differentiated information from the curricular snapshot displayed in the maps, e.g. regarding phases of progressing role integration. By combining both approaches, the status of role adoption can be diagnosed. On this basis, potentials for future curricular development and strategic decisions can be derived. Furthermore, NKLM reviewers might gain insight in critical parts of catalogue content.Keeping these considerations in mind, the following guiding questions are addressed: How do the implicit and explicit role patterns characterize the status of curricular integration of intrinsic roles? What strategies can be derived from these patterns for curriculum development and catalogue review?

## Methods

### Sample

To examine the curricular status quo of the intrinsic roles in a multi-site approach, six German medical faculties (Tuebingen, Freiburg, Ulm, Hannover, Bonn, and Magdeburg) participated in a joint NKLM mapping project [31, 32]. Led by the Competence Centre of University Teaching in Medicine Baden-Wuerttemberg in Tuebingen (CCMD), the mapping was conducted from summer 2016 to spring 2018. The faculties are characterized by structural, organizational and educational differences within the scope of governmental regulations guaranteeing minimum standards for UME. The programs’ general curricular status reveals a huge range of quantity of mandatory courses (98–177 courses per program) due to the heterogeneous granularity of organizational structure (teaching units). The developmental process for NKLM implementation didn’t start from scratch, because most faculties had already integrated special trainings for some competencies beforehand (e.g. communication, scientific-medical skills). In the present study courses covering five years compulsory programs of UME were mapped and analysed. On basis of previous project experiences, mapping of at least 80% of courses was considered as sufficient to ensure a reliable dataset for adequate characterization of intrinsic role patterns. So far, four programs achieved 95–100%.

### Data collection

To ensure standardized data formats and consistent data management, all institutions used the web-based MER*lin* mapping database as common mapping tool with protected proprietary data spaces. They also applied comparable consented procedures, as described in detail earlier [33, 34]. A short summary is given below. Courses were mapped against the given German competency-based framework NKLM [25] by selecting pre-set menu options [33]. The present analysis focused exclusively on the mapping data related to the intrinsic roles’ chapters (Chap.): Scholar (Chap. 6), Collaborator (Chap. 8), Health Advocate (Chap. 9), Manager (Chap. 10), and Professional (Chap. 11). The Communicator (Chap. 7) was excluded because of its reduced descriptive granularity, with the role being described only on competency level in the current version of the catalogue and its objectives relocated to other chapters. The following criteria were relevant for this study: (1) Objectives coverage: learning objectives being ticked off in case they were taught; (2) Transparency of teaching: characterized either by the term “explicit” (written in a study-guide, module manual or other course material), or “implicit” (implied, but not documented in writing). The term “mapping citation” referred to any objective taught in a course and ticked off by the mapper. Teaching an objective on one or more occasions in a course corresponded to one citation. For each site-specific program, the following quantitative data were collected from the database: total number of curricular courses and of general role citations, number of explicit and implicit citations for every role and number of related courses.

### Data quality control

In support of content validity, the mapping was conducted by 47–80 faculty members per program: individuals from each discipline or department, mostly preceptors with content-related expertise of courses or coordinating and/or supervising medical teachers with educational background. To harmonize the mapping process and data quality, curriculum mappers and local supervisors were trained and consulted by the CCMD individually and continuously. Plausibility controls of mapping data were carried out two-fold, by dean’s office and by responsible senior teachers coordinating departmental teaching. Consistency checks were done by the global administrator from CCMD.

### Data processing and visualization

To create a role-specific Matrix Map for illustrating the general curricular status of every intrinsic role, two criterions were selected: first, curricular weighting of the objectives, second, degree of multi-site agreement. The criterions were chosen because of their key relevance for curriculum description and availability of pre-existing usable multi-site mapping data. The process of calculation is described below.

#### Relative weighting of an objective

For intra- and inter-role comparison, the diversity of program sizes (number of courses) and the NKLM role chapter sizes (number of objectives, see legends of Fig. 2.1–5) were considered. To facilitate inter-role comparability of learning objectives, percentage shares of courses and program’s mapping citations were calculated. The percentage of citations expresses the curricular weighting of an objective, assuming that the more courses display a specific objective, the higher its curricular emphasis. To gain a general picture of curricular role representation, the mean weighting value was calculated for each of the objectives over the six programs. For roughly pointing out high or low weightings, a “general mean” was generated as an inter-role reference line. Multiplication of the above-mentioned average values by the total number of all roles’ objectives resulted in a common mean of 100 for all roles, thus facilitating easy inter-role comparison.

#### Extent of agreement between programs

To investigate the programs’ agreement, the percent agreement between the weighting values for each objective over the six programs was calculated on base of their average distance to each other. Each resulting value was subtracted from a reference scale with 100 representing full agreement: the lower the average distance between programs’ weightings, the higher the agreement. An inter-role reference line was set at 50 on this scale. Weightings and agreements were determined in the same way for both, the explicit and implicit mapping data.

#### Qualitative matrix map

To visualize the relationship between the roles’ curricular weighting and the extent of agreement, a 4-Field-Matrix was generated for each role, enabling a systematic analysis of value and risk potentials [27]. Supporting intuitive interpretation, the agreement values were applied to the x-axis and the weighting values to the y-axis. To rank the objectives of a role roughly, ordinal scales in ranges of “high” and “low” were used with the above-mentioned general reference lines as mid marking (midlines). The matrix position of an objective in one of the four quadrants was determined by its coordinates of weighting and agreement. Every objective is documented as a circle and can be identified individually. All role-specific average values of objectives were plotted into the matrix, resulting in two scatterplots (point clouds) per role, one with explicit (filled circle) and one with implicit values (empty circle).

Since this study was focused on transparency patterns of the intrinsic roles in general rather than on details, decisions were made for the benefit of clarity: (1) the very few weightings outpacing the general mean more than twofold, were placed at the top end of the scale not to lose sight of the whole picture; (2) objectives of each intrinsic role were not labelled in the matrices, although they can be identified individually. Only few selected anchor examples were accentuated to visualize the matrix positions of explicit and implicit values of an objective indicating their relation to each other. The criteria used to pick the examples were: role-typical feature, significant differences between explicitness and implicitness if applicable, relevant content, contribution to complete coverage of all quadrants. Their NKLM chapter code numbers and short titles are given to catch the meaning (cp. Table 1).

**Fig. 1:**
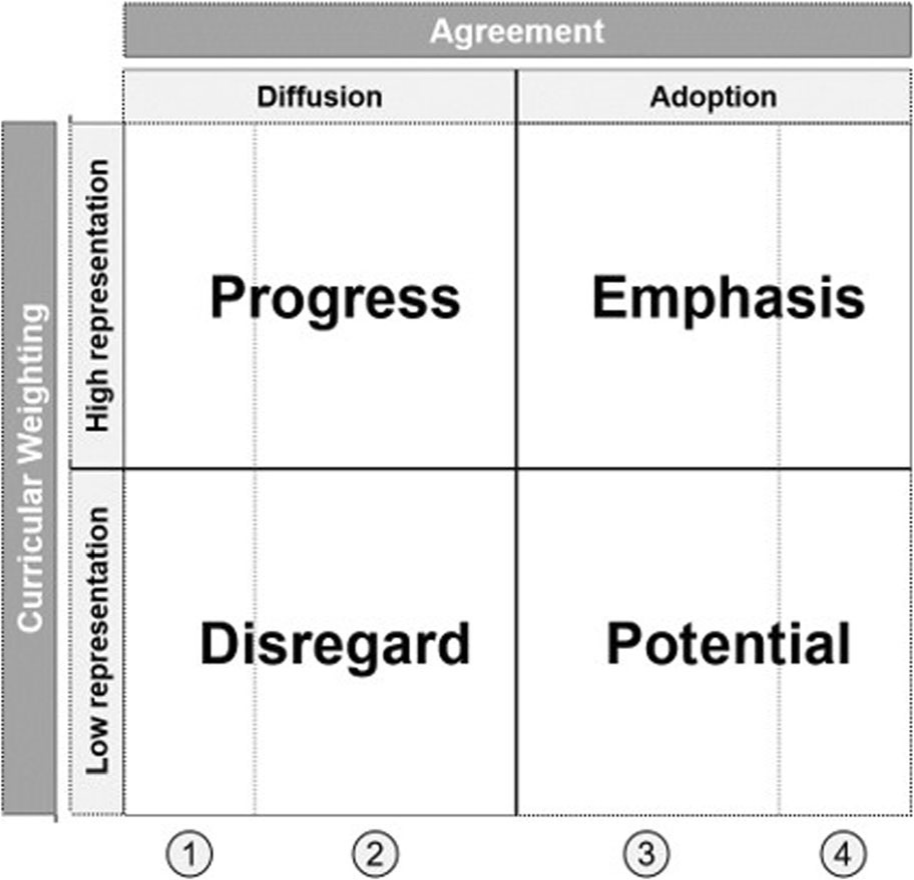
Adapted 4-Field-Matrix Map. The four quadrants are formed by interlacing curricular weighting and multi-site agreement (dark grey) and adding midlines. Rogers (1995) stages of diffusion are integrated (from left to right): 1. Knowledge-building & Persuasion, 2. Decision, 3. Implementation, 4. Confirmation. The vertical lines in the Matrix Map symbolize the transition between the stages (from left to right): Tipping point, Shake out, Saturation

To facilitate data interpretation in the context of change, the quadrants of the matrix were labelled after an extensive literature research of characteristics and application areas of Matrix Map Methods [27–29] and Rogers’ Model of Diffusion [30]. Following Rogers model, further reference lines were integrated from left to right in the matrix to indicate the four normally distributed main stages and adopter categories: (1) Knowledge-building and Persuasion (Innovators and Early Adopters 16%), (2) Decision (Early Majority 34%,) (3) Implementation (Late Majority 34%) and (4) Confirmation (Late Adopters 16%) (cp. Fig. 1). For each quadrant, optional strategic actions were weighed with reference to literature, based on exemplary objectives aiming at lead strategies and measures (cp. Table 3). Quadrant labels and strategic choices were discussed in the project group and adapted to the given situation of framework implementation.

#### Statistics

All statistical calculations were conducted using the standard softwareSPSS, Version 25, and Excel, Microsoft Office Version 2010.To test for intra-role differences between explicit and implicit curricular representation, the Related-Samples Wilcoxon Signed Rank Test was used.

## Results

For better clarity and comprehensibility, the results are presented in two steps: first, the concept of the adapted Matrix Map Model is explained with its informative value; second, the specific patterns of the intrinsic roles displayed in the given grid are described. Analyses of map characteristics reveal the current role status, which imply different strategic recommendations.

### Matrix map model

The 4-Field-Matrix was constructed based on the multi-site mapping data of the intrinsic roles. As result of the methodical discussions, two key dimensions were linked together to form a model: first, curricular weighting of role’s objectives, indicating the amount of representation in courses; and second, extent of programs’ agreement, both ranging from low to high (Fig. 1). According to Rogers (1995) [30], the left side of the matrix was defined as diffusion period covering the stages from knowledge-building to decision. The midline symbolizes some sort of shakeout of learning objectives between diffusion and adoption. From that point onward a clearly recognizable program consent is achieved. The right side was related to the adoption period including implementation and confirmation until even late adopters participate (maturity and saturation).

The amount of curricular weighting expresses the degree of value given to specific objectives, assuming the higher the course addressing a learning objective the higher its appreciation. The corresponding midline is formed by the average weighting of objectives across all roles (general mean). Given that orienting coordinate cross, the field is divided in four quadrants, as listed in an arc from lower left to lower right: Disregard, Progress, Emphasis and Potential.

The Disregard quadrant (lower left) contains objectives with apparently lower curricular appreciation but also with less consistency between the programs. Here lies content that isn’t paid much attention to until now. This may be because it is newly developed, experimented with or introduced at some sites in small scale. Simultaneously there is content located, that is disregarded for being unworthy of being depicted in UME. For objectives in that quadrant, it is highly uncertain if they will persist, develop, or perish, depending on the number of programs adopting them in future.

The Progress quadrant (upper left) is characterized by objectives showing high curricular presence, but only in some programs representing great diversity of site-specific local views. Thus, there is already high value and attention given to these objectives. Objectives in that field are obviously on the move, but the extent of consolidation appears yet to be undecided.

The prospects are high that these objectives are strengthened by further diffusion. It will be decisive to achieve a critical mass and cross the tipping point (chasm) between persuasion and decision stage in order to reach an early majority and enable larger adoption. Nevertheless, there is a possibility for innovative programs that their example is not followed by the majority; thus, it is an option to build up local profiles as standalone solutions or to give up that position.

Objectives located in the Emphasis quadrant (upper right) are characterized by high curricular weighting and adopted by the majority of programs. This maturity level indicates that this content can be classified as well anchored in UME (implementation stage) until saturation is reached (confirmation stage). Although there is a possibility of over-estimation in weighting, these objectives can be considered as set and are not a matter of priority in curriculum development.

The Potential quadrant (lower right) is characterized by lower curricular weighting but high agreement between sites. Objectives in that quadrant appear to be settled as well, however to limited extent of representation. The localization may have different reasons: objectives may be in the right place, because they are of minor importance or only few courses are necessary to reach the intended outcome, being refined in postgraduate medical education (PME); objectives may also be underestimated by many, because the change in demand was not realized (blind spot), consciously ignored in its importance or acknowledged but not reacted on yet. Similar as in the Disregard field, there is a chance that content located here is misplaced in UME (e.g. handed on for traditional reasons).

### Display of role patterns

In the following, the patterns of the roles in the matrix will be described, referring to key aspects of overall positioning, shape, concentration and the relationship between explicit and implicit parts (Fig. 2.1–5). Thereby specific role characteristics are carved out, exemplified by selected representative objectives (anchor examples; cp. Table 1). The varying amounts of learning objectives in the roles were considered but turned out to be neglectable.

**Fig. 2:**
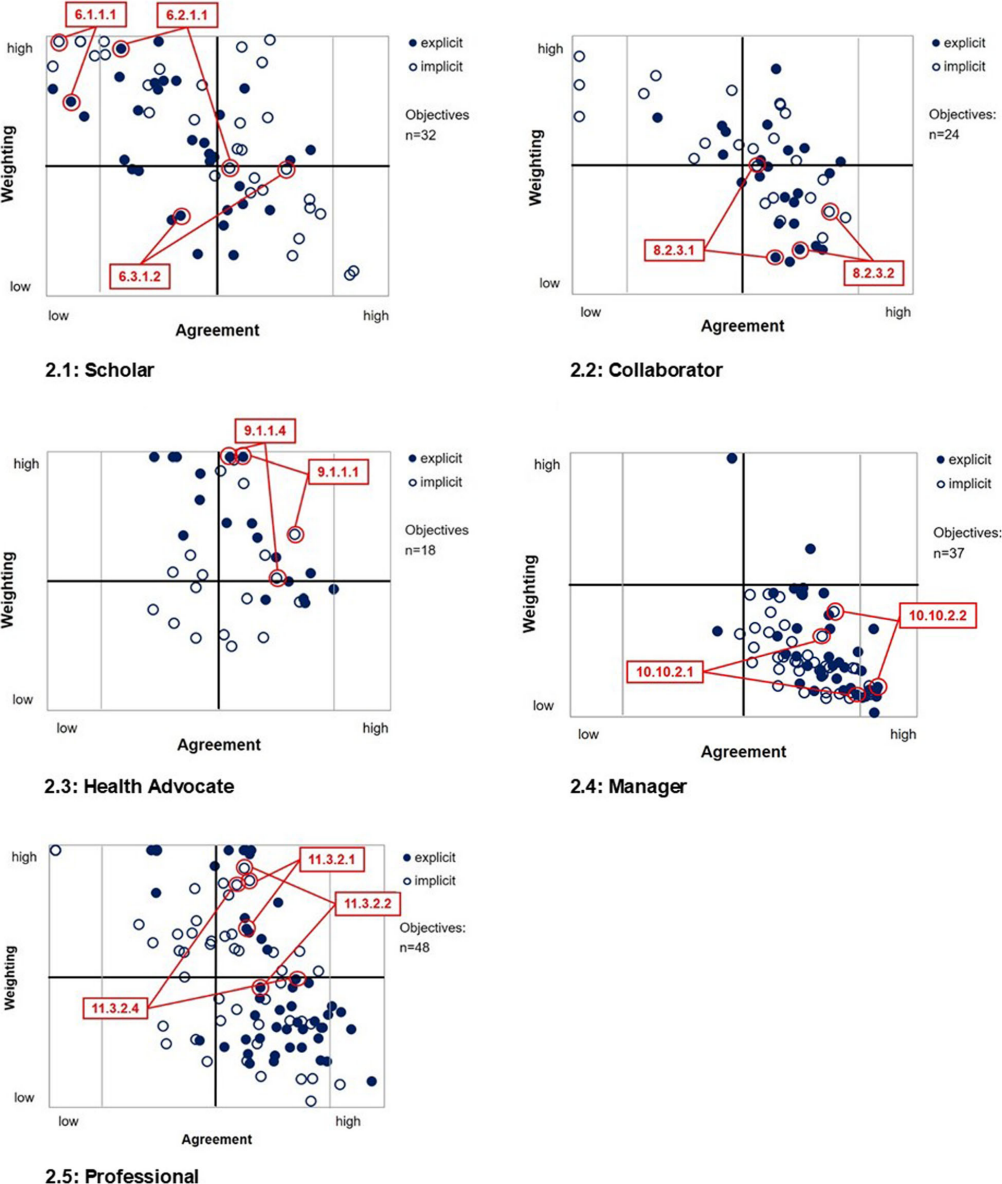
Role patterns in the 4-Field-Matrix. In the sub-diagrams 2.1–2.5 the explicit (filled circles) and implicit role patterns (empty circles) are displayed in Matrix Maps. Anchor examples are marked in the matrices with NKLM chapter code numbers linking implicit and explicit values of the objective/s. More detailed information is available in Table 1 (cp. column 3)

**Table 1:** Role-typical positioning in Matrix Map quadrants

Role	Quadrant	Chap. ID	Text	Weighting	Agreement
Scholar	Progress	6.1.1.1	Take responsibilities for continuing training, maintaining and developing competencies.	Explicit ➚ Implicit ➚ **exp < IMP**	Explicit ➘ Implicit ➘
	Progress Potential	6.2.1.1	Name and recognize quality criteria of scientific work and disturbing influences.	Explicit ➚ Implicit ➘ **EXP > imp**	Explicit ➘ Implicit ➚ **exp < IMP**
	Disregard Potential	6.3.1.2	Detect learning needs of students, peers and other health care professionals.	Explicit ➘ Implicit ➘	Explicit ➘ Implicit ➚ **exp < IMP**
Collaborator	Potential	8.2.3.1	Analyse interprofessional conflicts and reflect on perspectives.	Explicit ➘ Implicit ➘ **exp < IMP**	Explicit ➚ Implicit ➚
		8.2.3.2	Contribute to constructive solutions of interprofessional conflicts.		
Health Advocate	Emphasis	9.1.1.1	Integrate prevention as key elements into individual care.	Explicit ➚ Implicit ➚ **EXP > imp**	Explicit ➚ Implicit ➚
		9.1.1.4	Identify individual resources for improving the individual situation.		
Manager	Potential	10.10.2.1	Identify different leadership styles and management tasks, reflect on effectiveness.	Explicit ➘ Implicit ➘	Explicit ➚ Implicit ➚ **exp < IMP**
		10.10.2.2	Assume management responsibilities in a team (e.g. in study- or work groups).		
Professional	Emphasis	11.3.2.1	Observe and critical reflect on themselves.	Explicit ➘ Implicit ➚ **exp < IMP**	Explicit ➚ Implicit ➚
	Emphasis Potential	11.3.2.2	Realistically assess own competencies and take on tasks accordingly.		
	Emphasis	11.3.2.4	Express, accept and reflect on criticism and change behaviour accordingly.		

Selected anchor examples (Explicit and Implicit learning objectives) enable a more detailed look on the roles’ developmental status. Weighting and agreement are given with tendencies Texts are given in shortened version

Without differing between explicit and implicit, in an overall assessment of scatterplots, most intrinsic roles revealed a quite similar spatial orientation, being condensed primarily in the Progress and Potential quadrants with mainly one-sided low numbers. In case objectives are positioned in the Emphasis or Disregard quadrants, they mostly appear close to the adjacent quadrants.

Regarding the implicit-explicit relation the roles exhibit a more differentiated picture. The general analysis of the intrinsic roles revealed a tendency of implicit citations being higher than the explicit ones, but no significant differences were detected, except for two roles: The Collaborator displays higher implicit than explicit mapping data (z = − 3.657; *p* < .001), whereas the Health Advocate reveals higher explicit than implicit citations (z = 3.724; *p* < .001). In this approach the first focus is set on explicit depiction, with the implicit depiction serving as a supportive element for the estimation of factual but potentially hidden values of objectives.

The Scholar’s explicit objectives are broadly scattered across the whole matrix (Fig. 2.1), being clearly the role with the most objectives concentrating in the Progress quadrant. This shows that greater parts of the Scholar role, especially regarding the scientific competencies have started evolving visibly in an increasing number of programs (Fig. 2, Table 1).

Only in that role, some objectives are found in the Progress quadrant in a phase of early diffusion indicating rising attention (knowledge-building and persuasion). This is the case for aspects of lifelong learning. Explicit objectives being in the decisive stage are represented stronger than in any other role. In the adoption period, the number of Scholar’s implicit objectives are predominant compared to the explicit ones, showing higher agreement on implicit parts between sites. This concerns above all elements of teaching positioned explicitly in the Disregard and implicitly in the Potential quadrant. Overall, greater parts of the implicit curriculum reach equally high expression as the explicit with respect to curricular weighting.

In contrast to the Scholar, the Collaborator and the Health Advocate shows more explicit representation of objectives in the adoption period, revealing a higher level of maturity (Fig. 2.2, 2.3). The difference between the role depiction of Health Advocate and Collaborator lies in the extent of explicit curricular weighting opposite to the implicit counterpart showing an inverse relation: The objectives of the Health Advocate are obviously met with high explicit weighting by the majority of programs, still displaying signs of transition (early and late majority). Nevertheless, a greater part of explicit objectives was positioned in the Emphasis quadrant, e.g. regarding sub-competencies (SC) of prevention and individual health care (SC-9.1.1.). The explicit curriculum exceeds the implicit in every aspect. Unlike for the Health Advocate, the Collaborator’s explicit objectives appears clearly lower in weighting, being positioned mainly in the Potential quadrant. This indicates that a larger majority of programs already agreed upon this dimension of curricular representation (e.g. SC-8.2.3: Recognize interprofessional conflicts and actively contribute to solutions). However, in this role, the implicit curriculum exceeds the explicit clearly, reaching up to Progress or Emphasis quadrant. This finding reveals the existence of high but also informal and inapparent esteem for the Collaborator (risk of hidden curriculum).

The Manager role is cumulated exclusively in the Potential quadrant concerning both the explicit and implicit pattern (Fig. 2.4). The Manager is the only role to exhibit very low explicit curricular weightings for a larger set of objectives. Most of the programs obviously share a common view on the current value of the Manager. The role is in a stage of consented curricular implementation indicating a certain level of saturation. Some of its objectives possibly range on the brink to decline. There are currently no distinct signs of development visible.

Visualizing the described intrinsic roles spectrum, the Professional role shows an in-between status (Fig. 2.5). As whole, its explicit curriculum appears to be well-adopted in most programs, with the majority of content at the stage of maturity. However, the explicit objectives are settled primarily on lower level in the Potential quadrant with single high-weighted clusters in the Emphasis and the Progress quadrant. The implicit objectives are dominating in those upper quadrants with higher curricular weighting, e.g. for aspects of self-reflection and self-development (SC-11.3.2). This considerable excess of implicitness in the Progress and Emphasis quadrant signals a high degree of perceived relevance but also of non-transparency.

## Discussion

Originally medical roles were developed with the aim of “explicitly re-framing traditional implicit understandings of physician competence” [1]. But the integration of intrinsic roles into educational reality proved to be problematic. Our multi-site study in German contexts shows that it would be worth to deliberately and cooperatively evaluate the implementation of intrinsic roles from the beginning [35]. In the phase of implementing changed national recommendations, quick and reliable orientation in the field is especially relevant for curriculum planners and framework reviewers alike. Coming to informed decisions that fit the graduate profile, is obviously not a self-running process in role implementation. The relevance of our flexible approach becomes even more obvious, when considering the provisional and theoretical character of frameworks that continuously needs refinement [36].

### Application of the matrix map model in role implementation

Matrix Maps are widely used in economics in different appearances, acting as a guiding tool to categorize and initiate strategic measures [27]. Today adjusted, non-prescriptive matrices are required for practitioners to accompany experimentation and planning processes, as well as to accelerate decisions in an unpredictable and dynamic field. Characteristics of appealing matrix maps are in this context: catchy labelling, performance enhancement (e.g. consented weighting), universal applicability and interpretive space (e.g. combination with other tools) [28, 29].

As shown in this study, the approach is adaptable to needs of UME change by considering knowledge and techniques from areas like curriculum development, change and project management (e.g. identifying critical role parts, prioritizing interventions, and planning curricular revisions) [8, 23, 37].The Matrix Map Method provides a simplified but clear overall insight in the current developmental state of roles by entangling two factors relevant in national programs (curricular weighting, programs’ agreement). Advantages and disadvantages are compactly contrasted in Table 2.

**Table 2:** Advantages and disadvantages of the methodical approach

Advantages	Disadvantages
Multi-site evidences	Dependency on site-specific aspects
Holistic overview	Lack of detail
Reduction of complexity	Selection of criteria
Structuring (categorisation, prioritization)	(Possible) limited accuracy
Vivid depiction (intuitive usability)	Snapshot
New perspectives (impulse)	Lack of clear-cut solutions
Established method (in other contexts)	Unclear cultural fit (in medical context)

By illustrating different stages of role adoption as well as unveiling the relationship between implicit and explicit data, our methodical approach opens new perspectives on the implementation process of roles. More differentiated quadrant-specific interpretations can be derived for curriculum improvement by considering the implications of the stages of innovation diffusion [30]. Tailored to the quadrant characteristics and needs (described in results), a pre-defined lead strategy is assigned to each matrix quadrant specifically, indicating how to generally deal with the role parts located there by applying adequate measures (Table 3). Thus, reducing a multi-faceted matter in complexity, the conclusive pictures support strategic discussions for site-specific curricular orientation and evaluative catalogue review from a holistic perspective. Since objectives may not all be equally relevant for a compulsory core curriculum at undergraduate level, the overall aim requires to achieve a higher explicit agreement between faculties. This applies regardless of high or low curricular representation. All in all, in the German UME contexts, the steps (analysis, appraisal, strategic decision) are comparable to the original field of application in economics.

**Table 3:** Quadrant-specific lead strategies and measures for curriculum development and catalogue revision

Quadrant	Diffusion Stages	Strategy	Priority	Measure choices	Role examples
Disregard	Knowledge-building Persuasion Decision	Decision Strategy	CurD *	Awareness Well-dosed development (reduced?) Consenting between sites	Scholar
			CatD **	Compilation/reformulation Promoting Abandoning	
Progress	Knowledge-building Persuasion Decision	Development Strategy	CurD ***	Profile-building Disseminating (best-practice) Consented level adaption	Scholar Health Advocate
			CatD **	Monitoring (multi-site development) Estimation Re-calibration	
Emphasis	Implementation Confirmation	Assurance Strategy	CurD **	Monitoring Safeguarding (maintain status) Balancing/sharing	Health Advocate Professional Collaborator
			CatD *	Awareness (must-haves) Keeping track (topicality?) Confirming	
Potential	Implementation Confirmation	Evaluation Strategy	CurD **	Keeping track (change?) Emphasizing Consolidating	Manager Professional Collaborator
			CatD ***	Evaluating status Promoting Re-locating (PME)/Abandoning	

*CurD* = Curriculum developers, *CatD* = Catalogue−/Framework developers

*** = higher priority; ** = medium priority; * = lower priorit*y*

### Strategic implications of explicit role patterns

In the following, the proposed strategies and measures are discussed based on the analysis of the role patterns. The focus is on the roles’ explicit localization in quadrants. By using a typical role as an example from practice, the characteristic strategy of a defined quadrant is described with reference to literature. Detailed measures for curriculum developers (CurD) as well as catalogue reviewers (CatD) are presented in Table 3. The quadrants are addressed going backwards on the arc of diffusion, starting with established roles in the right quadrants (Potential, Emphasis) to evolving ones in the left ones (Progress, Disregard).

The Manager role is found almost exclusively in the Potential quadrant. The compactness of the role signals stable positioning in this quadrant, in the first place because of the high agreement of faculties. Nevertheless, the positioning of the role indicates the need to evaluate the overall minor curricular representation of all objectives (Evaluation Strategy). Nowadays, managing and leading competencies and key topics such as health economics, healthcare strategy, human resource and process management are demanded to be introduced as an integral part into UME, ideally by addressing these topics at basic competency level to advanced medical students in their clinical years [38, 39]. Thus, objectives in that field recommend an alert awareness for the lower curricular depiction and the environmental development. Further measures might be necessary like emphasizing particular topics regarding e.g. exposure time, level and performance quality, moving objectives to the Progress or Emphasis quadrant. However, if an objective is considered to be misplaced, another option might be re-locating it e.g. to PME. The Potential quadrant should be addressed with medium to high priority.

The Health Advocate displays a picture of agreed moderate to high curricular weightings at many sites. A greater share of its explicit objectives is positioned in the Emphasis quadrant, outweighing their implicit counterparts. This special pattern points out key elements that need lasting consolidation (Assurance Strategy). It may be explained with previous high political pressure and change in regulations in Germany [40]. Internationally, it is commonly consented that integrating advocacy compulsorily in UME is essential [41–43]. Thus, numerous curricular interventions were developed recently varying in timing, frequency, and intensity across the undergraduate curriculum [43]. Nevertheless, there is an “ongoing debate about whether physicians have a moral, social and professional responsibility to address health care issues beyond the direct care of their patients” [42, 43]. On this basis, it will be necessary in the next years to evaluate throughout, which elements of the fledgling curricula meet the needs of future physicians best, and in the long run patients and communities [44]. However, for now these obvious must-haves located in that quadrant need to be monitored, and measures to safeguard the reached status quo need to be taken. The Emphasis quadrant is of lower priority for developmental measures.Besides the Health Advocate that shows several high ranked and evolving explicit objectives, a greater part of the Scholar is located in the Progress quadrant. Objectives in that quadrant are in a transitional phase. Faculties’ readiness for change and targeted experimentation determines the diffusion of promising concepts at this early stage (Development Strategy). In Germany, scientific competencies were newly focused as explicit educational needs with the NKLM catalogue [25, 34]. The example (Fig. 2.2) shows that especially the international demand for adequate scientific education and lifelong learning increasingly caught attention of many faculties, following political promotion. Many institutions recently developed curricular interventions, covering some objectives with a higher curricular weighting, although with lower agreement between sites [34]. The main strategic focus for that field is to consciously prioritize and decide on the degree of curricular weighting and external impact, thus moving objectives to the Potential or the Emphasis quadrant. The decision about further development of objectives in that field is primarily depending on local criteria (e.g. urgency, resources, context, local profile). Objectives in that quadrant should be handled with higher priority.

Currently, the Scholar is the only role with explicit objectives being clearly situated in the Disregard Quadrant. In this quadrant, “unbeloved” facets of the intended graduate profile and small-scale interventions are found. These objectives should be in the focus of NKLM review, as informed stay-or-exit decisions on objectives in that quadrant of uncertainty are needed (Decision strategy). The Scholar, consisting of three different dimensions (scientist, learner and teacher), appears as a torn role regarding curricular attention and representation) [3, 7, 45]. While some parts are in progress or well set, especially the explicit integration of teaching aspects (e.g. peer tutoring) in curricula is comparatively underrated or not depicted (enough); even though this aspect might be a helpful add at many stages in the curricula (e.g. reduction of resources, build-up of competencies).

### Impact of the implicit-explicit relation of roles

Besides combining the matrix map with the diffusion stages, an additional source of information with argumentative power is provided by integrating implicit patterns in role’s status diagnosis. The extent of implicit and explicit overlap revealed different degrees of matching, indicating salient discrepancies of curricular transparency. Particularly concerning roles like e.g. the Collaborator with implicit patterns clearly exceeding the explicit, a profit can be drawn from this matrix illustration (Fig. 2.4). As sketchily outlined, the implicit pattern concentrates in the Progress quadrant, whereas a greater proportion of the explicit pattern is settled in the Potential quadrant. The visible gap in the Collaborator role, underpinned by the statistical analysis, indicates the existence of a hidden curriculum, potentially undermining the theoretical specifications of the framework. Empirical data from literature support this finding: In line with international postulations [41], great efforts have been undertaken to emphasize the role’s competencies [46]. At the same time, educational interventions were often hindered by hidden or informal curricula [47, 48].

The Professional role is another role obviously with a higher implicit weighting of objectives signalling non-transparency and informality of teaching (Fig. 2.5). Professionalism is often seen as an invisible competence that is always present in medical education, but not always explicitly addressed [18]. Although there is no consent about the best way to teach professionalism in medicine, all methods that have shown being effective are personalized and primarily implicit: role modelling, mentoring and critical reflection [49–51]. With respect to the high relevance in clinical practice, it is important to be aware of the implicit nature of the Professional and to consider the danger of underestimation or blind spots. Teaching and assessment of medical professionalism, notably reflection and feedback as driving forces for competence development, need to be explicitly rooted in UME in main features. Reinforcement and internalization have to be entrusted to the individual when gathering experiences in practice [50, 51].

Therefore, especially roles with higher weighted implicit shares need to be carefully evaluated to recognize the existing implicit values of objectives. This forms the fundament for reliable curricular integration and constructive negotiations about resource allocation in the faculties. Catalogue reviewers need to adapt guidelines for these roles in discussions with the faculties and professional societies. Homogenous role compilation and perception is required to consent the degree of needed explicitness and to refine quadrant-specific strategies. Generally, as discussed for the Professional, the tendency to explicate more of the implicit has to be strengthened and thereby moving objectives to the Emphasis quadrant [52].

### Limitations

The methodical approach with using mapping data in focused matrix maps is a good basis but has some restrictions. Despite the obvious strengths of the present approach (e.g. multi-site data reference, curricular overview, evidences for informed self-assessment), some further aspects have to be considered. The role patterns are a crosscut in the continuously changing field of UME, flexible re-checking at other points of time are necessary to visualize longitudinal developments. They cannot be taken as hard data and accurate values, describing the curriculum precisely. Over−/underestimations of competency representation cannot be excluded despite mappers’ instruction. Mapping data may be negatively biased among others by lack of knowledge, framework terminology, misperception of intrinsic roles as well as general minor CBME acceptance and an unfavourable status of change in teaching culture [5–7, 23]. Because of grown curricular differences of the participating faculties, a mathematical relativization of mapping data is necessary to enable comparability. Although teachers’ perspectives on the explicit and implicit curricular performance of the intrinsic roles are highly relevant, there is still a risk of an unnoticed informal and hidden curriculum. Students’ mapping, as a controlling perspective, is relevant for quality assurance, but is addressed in another project part.

## Conclusion

The flexible 4-Field-Matrix, as applied in our study, enables to catch the status of intrinsic roles’ implementation in German UME at a glance. Our findings reveal clear role profiles regarding role pattern, implicit-explicit relation and programs agreement, thus forming a pragmatic and supportive knowledge base for differentiated interpretation. The illustration genuinely fosters informed discussions in both, catalogue revision and local curriculum development. Due to its complex, multi-faceted educational features of intervention, CBME depends heavily on characteristics of local culture and contexts, such as educational resources and traditions, faculty attitudes and institutional readiness for change [35]. Starting from the overview matrix, targeted analyses of mapping data can be performed tailored to institutional needs and goals in priority, scope and depth of detail. Strategically strengthening relevant objectives by framework and curriculum developers may help to build up explicit competency standards – adequate and commonly agreed. In the long run, making more of the implicit explicit enhances curricular transparency and facilitates the development of a teaching culture better fitting to CBME.

## Data Availability

The datasets analysed during the current study are not publicly available due to contract agreement. Data are available from the corresponding author in anonymized version upon reasonable request and with permission of the participating medical faculties as third party and owners of their local curricular data.

## Abbreviations

CBME: Competency-based medical education
CCMD: Competence Centre Medical Didactics (short form of: Competence Centre for University Teaching in Medicine, Baden-Wuerttemberg)
NKLM: National Competency-based Learning Objectives for Undergraduate Medical Education
PME: Postgraduate medical education
UME: Undergraduate medical education
